# SCLC extensive disease – treatment guidance by extent or/and biology of response?

**DOI:** 10.1186/1748-717X-3-33

**Published:** 2008-10-02

**Authors:** Franziska Eckert, Arndt-Christian Müller

**Affiliations:** 1 Department of Radiooncology, Eberhard-Karls-University Tübingen, Germany

## Abstract

In extensive disease of small cell lung cancer a doubling of the one-year-survival rate was reported in August 2007 by prophylactic cranial irradiation applied to patients who experienced any response to initial chemotherapy. We discuss the treatment concept of extensive disease in the face of the latest results and older studies with additional thoracic irradiation in this subgroup. A randomized trial with prophylactic cranial irradiation published in 1999 demonstrated an improvement of 5-year-overall-survival for complete responders (at least at distant levels) receiving additional thoracic radiochemotherapy compared to chemotherapy alone (9.1% vs. 3.7%). But, these results were almost neglected and thoracic radiotherapy was not further investigated for good responders of extensive disease. However, in the light of current advances by prophylactic cranial irradiation these findings are noteworthy on all accounts. Considering both, a possible interpretation of these data could be a survival benefit of local control by simultaneous thoracic radiochemotherapy in the case of improved distant control due to chemotherapy and prophylactic cranial irradiation. Furthermore the question arises whether the tumor biology indicated by the response to chemotherapy should be integrated in the present classification.

## Background

Extensive disease of small cell lung cancer (ED-SCLC) is still a therapeutical challenge. The median survival time without treatment reaches two to four months [1]. Standard therapy with four to six cycles of chemotherapy prolongs the median survival time to six to nine months. Even with the achievement of a complete response (CR), only a few months more are added to the short life expectancy.

The investigations of the last years mainly concentrated on more effective and less toxic drug regimens or on targeted therapies, but until now these attempts did not bear essential success. Astonishingly, a doubling of the one-year-survival rate was reported in August 2007 for a "conventional" technique: Prophylactic cranial irradiation (PCI) applied to patients who experienced any response to the initial chemotherapy [2].

### PCI and ED-SCLC

For the first time an improved survival was shown for PCI by the mentioned EORTC-study in this favorable subgroup (at least partial response to chemotherapy). The bottom line was an increase of overall survival (27 vs. 13% one-year survival) based on improved local control with a highly significant reduction of symptomatic cerebral metastasis for patients treated with PCI (41 vs. 17%). With the shortest of the applied radiation regimens this meant a gain in overall-survival of six weeks (5.4 vs. 6.7 months median survival) with a therapy of one week. The assessed quality of life was significantly better in the radiation group due to acceptable toxicities and reduced morbidity caused by cerebral metastasis. An additional advantage of PCI and related lower morbidity was the increased applicability of second-line chemotherapy. The study was designed with focus on easy feasibility and cost-effectiveness. So, patients with symptomatic brain metastases were excluded and cross sectional imaging was not routinely demanded but performed in case of defined key symptoms. However, treatment of occult brain metastasis by PCI cannot be finally ruled out. Despite of this potential limitation, an older meta-analysis evaluating complete responders of SCLC illustrates the main principle that PCI improves survival (benefit of 5.4% at 3 years) [3]. Approximately 20% of complete responders included in this meta-analysis were staged as extensive disease. The subgroup-analysis revealed no significant difference between limited and extensive disease (relative risk of death for limited/extensive SCLC: 0.85 vs. 0.77 p= 0.88).

Local recurrence plays an increasing role in ED-SCLC. After thoracic radiation treatment, in-field relapse occurred in 24% of the cases as the first site of relapse [4]. Without local treatment 89–93% patients suffered from local progress in the first year after primary therapy [2].

### Additional local radiotherapy for extensive disease?

Already in 1999 a study performed by a Serbian group [5] evaluated different local treatment approaches depending on response to initial chemotherapy. Those who had complete response at least at distant levels were randomized to receive either two courses of cisplatin/etoposide (each 80 mg/m^2^) or TRT with daily carboplatin/etoposide (each 50 mg/m^2^). Subsequently, PCI and two more courses chemotherapy were performed. Interestingly thoracic radiochemotherapy improved 5-year overall-survival compared to chemotherapy (9.1% vs. 3.7%).

Four older studies [6-9] investigating the value of TRT without standard PCI for extensive disease were unable to detect any benefit from treatment. However, besides absence of PCI, chemotherapy was applied sequentially and not concurrent with TRT. Furthermore, the limited case number of all four trials together (129 vs. 109 in the study of Jeremic et al.) suggests a lower statistical power of the negative studies. Staging deficits of the earlier investigations in the late eighties might also be assumed. A possible interpretation of these data on TRT could be a survival benefit of local control by simultaneous radiochemotherapy in the case of improved distant control due to chemotherapy and PCI. TRT has never been implemented into clinical treatment concepts for ED-SCLC. However, these findings are noteworthy on all accounts potentially improving the outcome of good responders with extensive disease.

## Conclusion

**Figure 1:**
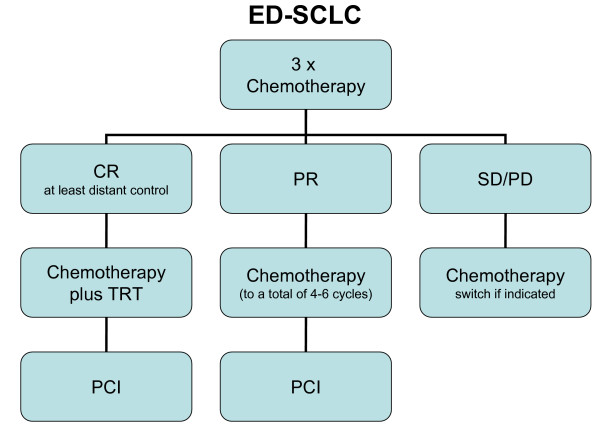
**Suggested treatment strategy for ED-SCLC**. Based on recent and older, hardly considered data [2,5] there could be three different treatment strategies according to initial response to chemotherapy: Chemotherapy plus TRT (simultaneously with the 4^th ^cycle) and PCI for good responders achieving complete remission at least at distant levels; chemotherapy and PCI for patients having any response; second line chemotherapy or best supportive care for stable or progressive disease.

Taken together the results of Slotman et al. and Jeremic et al. lead to the question whether the treatment for extensive disease SCLC should be reconsidered. There could be three different treatment strategies according to initial response to chemotherapy: Chemotherapy plus TRT (simultaneously with the 4^th ^cycle) and PCI for good responders achieving complete remission at least at distant levels; chemotherapy and PCI for patients having less than complete response; second line chemotherapy or best supportive care for stable or progressive disease (Figure 1). If this therapy was established, the difference in treatment of limited and extensive disease in complete responders would diminish. The best estimated 5-year-overall-survival for the described schedule could exceed 20% for limited and reach almost 10% for extensive disease [4,5,10]. Based on the available data the question arises, whether the present classification should be supplemented by biology of response. Surely, randomized trials are essential to evaluate this proposed procedure. Furthermore, the significance of potential confounders like treatment of asymptomatic brain metastasis by PCI, prognostic relevance of metastatic pattern within the heterogeneous group of ED-SCLC and subsequent second line treatment could be analyzed.

## Competing interests

The authors declare that they have no competing interests.

## Authors' contributions

FE drafted the manuscript, drafted the figure. ACM conceived the manuscript. All authors read and approved the final manuscript.
